# Advances and applications of organ-on-a-chip technology

**DOI:** 10.1016/j.crmeth.2026.101361

**Published:** 2026-03-23

**Authors:** Jeong Sik Kong, Jisoo Kim, Jinah Jang, Dong-Woo Cho

**Affiliations:** 1 Department of Mechanical Engineering, Pohang University of Science and Technology (POSTECH), Pohang, Gyeongbuk 37673, Republic of Korea; 2 POSTECH-Catholic Biomedical Engineering Institute, POSTECH, Pohang, Kyungbuk 37673, Republic of Korea; 3 Department of Creative IT Engineering, Pohang University of Science and Technology (POSTECH), Pohang, Gyeongbuk 37673, Republic of Korea; 4 School of Interdisciplinary Bioscience and Bioengineering, Pohang University of Science and Technology (POSTECH), Pohang, Gyeongbuk 37673, Republic of Korea

**Keywords:** organ-on-a-chip, microfluidics, bioprinting, injection molding, disease modeling, drug testing, personalized medicine

## Abstract

Advancements of the organ-on-a-chip (OOC) technology have been at the forefront of multidisciplinary convergence, blending biology, engineering, and microfabrication. OOC systems recreate human organ functionalities on microfluidic devices and offer more accurate alternatives to traditional testing methods. In this review, we discuss the various fabrication methods such as microfluidics, bioprinting, and injection molding, which are vital for the development of this technology. We further highlight the capability of OOC devices to accurately simulate human organ conditions and their applications in disease modeling, drug testing, and personalized medicine. The integration of OOCs in biological research, including as biosensors for real-time monitoring and in disease modeling, and the use of OOC systems in space research, particularly for studying the effects of microgravity and radiation on human health aboard the International Space Station, are also discussed. This technology shows immense promise for transforming approaches in drug discovery, toxicology, and personalized medicine.

## Teaser

Organ-on-a-chip technology recreates key structural and functional features of human organs within microfluidic platforms, providing physiologically relevant alternatives to conventional models. In this review, Kong et al. summarize the various fabrication and cultivation strategies and discuss their applications ranging from biosensors, drug testing, and disease modeling to space biology.

## Introduction

Organs-on-chips (OOCs) represent a rapidly advancing field of research that aims to recreate the physiological and functional characteristics of human organs on microfluidic devices.^1^^,^^2^ These devices, which are often referred to as “chips,” incorporate living cells, such as human organ cells or tissue constructs, in a controlled and biomimetic environment.^3^^,^^4^ OOC technology focuses on replicating the key features of an organ, including its structure, cellular composition, and dynamic microenvironment, in a compact and simplified form. This technology enables researchers to simulate and analyze the behavior and responses of human organs in a highly controlled and customizable manner.

OOC devices typically comprise microfluidic channels that mimic blood vessels or other fluid flow systems in the human body.^5^ These channels are lined with living cells that accurately represent organ functionality. By introducing various mechanical forces, chemical gradients, and fluid flows, researchers can recreate the complex interactions and physiological conditions that occur within specific organs.

Conventional 2D cell culture has been a cornerstone of *in vitro* research for decades. In this method, cells are grown as monolayers on rigid plastic or glass surfaces, which enables straightforward observation and high-throughput experimentation. However, this approach fails to replicate the 3D architecture of human tissues, which is essential for cell-cell and cell-matrix interactions. *In vivo*, cells exist in a dynamic microenvironment with gradients of oxygen, nutrients, and mechanical forces—features absent in static 2D systems. A critical drawback of 2D models is their inability to mimic tissue-specific mechanical cues. For instance, endothelial cells in blood vessels experience shear stress from fluid flow, while lung alveolar cells undergo cyclic stretching during respiration. The absence of these stimuli in 2D cultures leads to aberrant cell behavior, such as altered gene expression and drug metabolism.^6^ Furthermore, 2D systems lack functional vasculature, limiting studies on drug permeability, immune cell trafficking, and metastasis. This simplification undermines their utility in modeling complex diseases like cancers or neurodegenerative disorders, where stromal interactions and vascular dynamics are pivotal.

Animal models have long been the gold standard for preclinical studies, providing insights into systemic physiology and organ-organ crosstalk. Nevertheless, interspecies differences in genetics, metabolism, and disease mechanisms frequently lead to translational discrepancies.^7^ These disparities result in poor extrapolation of drug efficacy and toxicity data. Ethical concerns also plague animal testing, particularly for invasive procedures or chronic studies.

OOC technology bridges the gap between 2D cultures and animal models due to the use of human cells, improved physiological relevance through the replication of human organ conditions, better recapitulation of organ complexity through the incorporation of multiple cell types and tissue architectures, and precise control over experimental conditions for reproducibility; moreover, it offers several advantages, including, high-throughput screening of drugs, reduced reliance on animal testing, and higher potential for personalized medicine approaches.^2^^,^^8^^,^^9^^,^^10^ These advantages make OOCs promising approaches for advancing biomedical research, enabling more accurate studies of human organ behavior, and transforming aspects of healthcare and pharmaceutical industries. However, it currently faces significant limitations that prevent it from fully replacing animal testing due to challenges in recapitulating systemic complexity and immune system integration^11^^,^^12^

The field of OOCs is highly interdisciplinary, incorporating expertise in biology, engineering, materials science, and microfabrication. Researchers are continuously developing and refining these devices to mimic the complexity and functionality of various organs such as the liver, heart, lung, kidney, and brain. As technology progresses, OOCs continue to advance, demonstrating the potential to revolutionize drug discovery, toxicology testing, and personalized medicine, ultimately leading to more effective and safer therapeutic interventions.

In recent years, the development of OOC technology has increasingly focused on comprehensively recapitulating the complexity of human physiology, with particular emphasis on three key aspects: multi-organ integration, real-time biosensing, and patient-specific disease modeling. Multi-organ platforms, which interconnect organ modules such as the liver, kidney, heart, and lung via vascularized microfluidic channels, now enable the simulation of systemic pharmacokinetic and pharmacodynamic profiles with clinical relevance.^13^^,^^14^^,^^15^ Notably, the qualification of human liver-chip models as drug-induced liver injury (DILI) assessment tools by the FDA’s ISTAND (Innovative Science and Technology Approaches for New Drugs) program in 2024 marks a significant regulatory milestone.^16^ Concurrently, the integration of electrochemical biosensors allows for non-invasive, real-time monitoring of parameters such as oxygen tension, pH, and cytokine secretion, enabling earlier detection of drug toxicity compared to conventional methods.^15^^,^^17^^,^^18^ Patient-derived cells are increasingly being utilized to construct personalized disease models in areas such as neurodegenerative diseases and cancers.^19^ Collectively, these technological advances are not only accelerating the replacement of animal models but also reducing early-phase clinical trial failure rates and expanding the application of OOC technology to novel fields such as space biology, thereby driving a paradigm shift in biomedical research and therapeutic development.

## Fabrication methods for OOC devices

### Soft lithography-based microfluidic technology

Microfluidic technology plays a pivotal role in the fabrication of OOC devices. It involves the precise manipulation and control of small volumes (10^−9^–10^−18^ L) of fluids at the microscale, typically ranging from microliters to nanoliters.^20^^,^^21^^,^^22^ Microfluidics is a powerful tool for creating intricate networks of microchannels within a chip, which are essential for simulating the fluidic environments of human organs.

**Figure 1:**
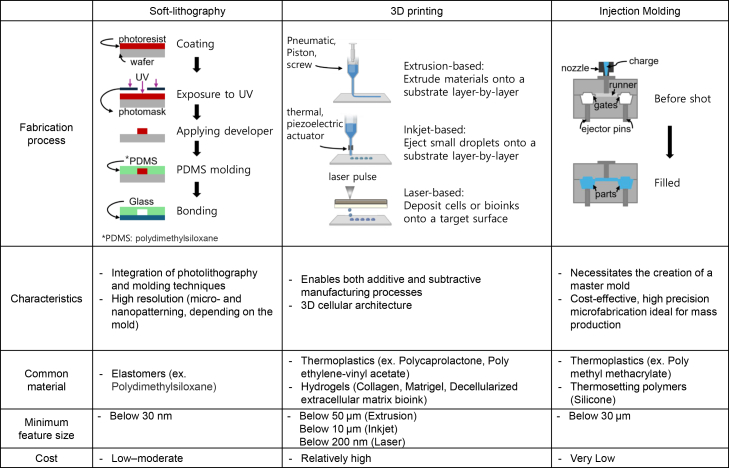
Common fabrication methods for organ-on-a-chip devices A comparison of the processes, characteristics, materials used, and costs of different methods, namely soft lithography, three-dimensional printing, and injection molding, is presented.

The fabrication of microfluidic channels often relies on techniques such as soft lithography.^23^^,^^24^ This process begins with the creation, using photolithography, of a mold, in which light-sensitive materials are selectively exposed to light through a mask to define the desired channel patterns. The mold is then used to replicate the pattern by pouring a soft material, such as polydimethylsiloxane (PDMS), over it. After curing, the PDMS replica is bonded to a substrate to form a microfluidic chip with the desired channel architecture (Figure 1).

Microfluidics enables fine control of fluidic conditions, including flow and gradient formation.^25^^,^^26^^,^^27^^,^^28^ This control allows researchers to replicate the dynamic fluid conditions observed in human organs, including blood flow, nutrient transport, and waste removal. By precisely regulating the flow of media, drugs, or chemical stimuli, researchers can create realistic microenvironments for cell or tissue constructs within a chip.^29^^,^^30^ Microfluidic channels can be functionalized with surface coatings or biological molecules to promote cell adhesion, migration, or differentiation. Different cell types have distinct requirements for surface properties, extracellular matrix (ECM) components, and biochemical cues. By selectively functionalizing different regions of the channels with cell-type-specific coatings, researchers can create optimal microenvironments that support the cultivation of various cell types within a single device, thereby mimicking the cellular composition of the targeted organ.^31^ The first functional organ-level microfluidic culture device, lung-on-a-chip, comprised two parallel hollow channels separated by a porous membrane coated with ECM.^32^ One side of the membrane was lined with human lung alveolar epithelial cells, whereas the other side was lined with human vascular endothelial cells, thus replicating the alveolar-capillary interface. The culture medium flowed through the endothelium-lined channel to simulate vascular perfusion, and air was introduced into the epithelial channel to mimic the air-liquid interface, which is crucial for lung differentiation and function. Cyclic tissue deformations were induced at the flexible tissue-tissue interface to mimic breathing motions by applying cyclic suction to the hollow side chambers. The ability to culture cells within microfluidic channels that are subjected to controlled fluid flows and chemical gradients enhances the physiological relevance of OOC models.

### Bioprinting technology

Bioprinting has emerged as a cutting-edge method for the fabrication of OOC devices.^33^^,^^34^ It enables the precise deposition of living cells, biomaterials, and bioinks to create 3D structures that closely resemble human tissues and organs.

Bioprinting utilizes specialized printers and bioinks, which are bioactive materials containing cells and biomolecules.^33^ The bioinks used in bioprinting are typically composed of hydrogels or other biocompatible materials that provide structural support and facilitate cell attachment, proliferation, and differentiation.^35^ These bioinks can be loaded with cells of interest, such as primary human cells, stem cells, or cell lines, which are carefully selected to mimic the desired organ or tissue. Thermoplastic materials such as polycaprolactone (PCL) and polyvinyl alcohol (PVA) are commonly used for fabricating 3D structural frames.

The fabrication process begins with the creation of a digital design or model of the OOC structure.^33^ The bioprinter then follows this design, precisely depositing the bioink layer by layer to build the 3D structure. The printers operate on principles such as inkjet-, extrusion-, or laser-based techniques.^36^^,^^37^ Inkjet-based bioprinters eject small droplets of bioink onto substrates layer by layer, whereas extrusion-based bioprinters extrude bioinks through fine nozzles. Laser-based bioprinters use laser-induced forward transfer to deposit cells or bioinks onto target surfaces. Laser-induced forward transfer (LIFT) bioprinting consists of a pulsed UV laser, a laser-absorbing layer with cell-containing hydrogel, and a receiving substrate. The system functions by focusing laser pulses on the absorbing layer (typically glass or quartz) to create vapor pockets that propel cell-laden droplets onto a substrate where they crosslink.^38^ Leveraging these techniques, researchers have fabricated OOC devices in a single step by using printing heads loaded with different materials, including biocompatible thermoplastics and cell-laden bioinks.^39^ The printed bioink can be crosslinked or solidified using various techniques such as UV light exposure or temperature control to stabilize the printed structure (Figure 1).

Improving physiological relevance, fabrication efficiency, and material versatility is crucial for developing advanced OOCs. In this context, bioprinting offers several significant advantages over conventional techniques. A notable aspect of this technology is its capacity to produce OOCs encompassing everything from a chamber to a range of cell-laden materials in a one-step fabrication process.^39^ Additionally, it enables the creation of complex and customized 3D tissue architectures, including vascular networks, tissue compartments, and organ-specific microenvironments.^40^^,^^41^ This process can precisely deposit cells and biomaterials in a controlled manner, enabling recreation of the cellular composition and spatial organization of human organs. Furthermore, the ability to select from a variety of materials highlights the immense potential of bioprinting for future applications. Bioprinting technology also facilitates the integration of multiple cell types within a single OOC device, replicating the heterogeneity observed in human organs.^42^ This capability facilitates the study of organ-organ interactions and complex physiological processes. Moreover, bioprinting technology enables the creation of tissues with functional features, such as blood flow, heartbeats, and CaOx crystal formation, using a multi-biofabrication technique with appropriate cell density and high cell viability.^43^^,^^44^^,^^45^ The printing process can be optimized to ensure cell survival and the maintenance of phenotypic characteristics, thereby enhancing the physiological relevance of OOC models.

Although bioprinting technology exhibits immense potential, challenges in terms of scalability and resolution in 3D cell patterning remain.^33^^,^^46^ Scaling bioprinted constructs to clinically relevant sizes while maintaining cellular viability and structural integrity presents significant challenges. As construct size increases, issues related to nutrient and oxygen diffusion become critical, particularly in the absence of functional vascular networks. Most bioprinting systems achieve resolutions in the range of 50–100 μm, which is inadequate for precisely controlling single-cell positioning or creating fine microstructural features essential for organ function.^47^ This limitation is particularly problematic for organs requiring precise cellular organization, such as kidney nephrons or liver sinusoids, where cellular spatial relationships directly impact function. Additionally, current bioprinting techniques struggle to create complex internal structures, such as branched vascular networks or hollow lumens, which are essential for developing physiologically relevant OOC models.^48^ Several approaches have emerged to enhance bioprinting resolution. Digital light processing (DLP) bioprinting has achieved remarkable improvements through the incorporation of iodixanol in bioinks, enabling a 10-fold reduction in light scattering and achieving 50-μm fabrication resolution, even at high cell densities of 0.1 billion cells per milliliter.^49^ Two-photon polymerization stands out as the most promising high-definition bioprinting technique, capable of achieving sub-cellular resolution below 50 μm and enabling truly 3D printing with unprecedented precision.^50^^,^^51^ Increasing production efficiency to address scalability requires systematic approaches to automation and standardization. Automated real-time quality monitoring and optimization during the printing process reduce manual intervention and improve reproducibility.^52^^,^^53^ Standardized bioink formulations and validated printing protocols are being developed to enable consistent results across different laboratories and manufacturing facilities.^54^^,^^55^^,^^56^

However, continuous advancements of bioprinting techniques, materials, and bioink formulations are rapidly addressing these challenges, driving the field of OOCs toward more realistic and functional models that mimic human organs and facilitate advanced research in drug discovery, disease modeling, and personalized medicine.

## Injection molding

Continued efforts to streamline microfabrication processes have led to casting and, more notably, the use of the injection molding technique.^57^ It offers precise and scalable production of microfluidic structures and enables the mass production of chips with consistent quality, making it ideal for commercialization. The injection molding process involves the use of a broad range of polymers, typically thermoplastics such as polycarbonate, cyclic olefin copolymer (COC), or polymethyl methacrylate (PMMA), in a mold cavity under high pressure.^58^^,^^59^^,^^60^ The mold is designed to replicate the desired microfluidic channels, chambers, and other features of an OOC device. The fabrication process begins with the creation of a master mold, which is typically fabricated using techniques such as photolithography or direct laser writing.^61^^,^^62^ The master mold contains the desired microfluidic channel patterns and is used to create replicas. The molten material is injected into the mold cavity, and it take the shape of the mold once it cools and solidifies. Once the material solidifies, the mold is opened, and the fabricated chip is removed (Figure 1). Lee et al. suggested an injection-molded plastic array 3D culture platform that forms patterns partitioned in a 96-well format. Using this OOC platform, a high-throughput 3D co-culture of human umbilical vein endothelial cells and lung fibroblasts was established to determine the angiogenic sprouting behavior.^63^ The injection molding technique also enabled the development of an easy-to-use 3D spheroid culture platform for anticancer drug efficacy testing by cultivating vascularized tumor cell spheroids.^64^ With their potential for mass production, these platforms offer convenient and readily usable solutions that are suitable for a wide range of experiments.

## Comparison of soft lithography, bioprinting, and injection molding

Soft lithography-based microfluidic technology, bioprinting technology, and injection molding technology each play distinct roles in the fabrication of OOC devices, particularly regarding their resolution and reproducibility.

Soft lithography is the most widely used technique for microfluidic OOC fabrication. It typically employs PDMS and photolithographically produced master molds to achieve high-resolution microfeatures. Soft lithography enables feature sizes as small as a few micrometers, with reports of sub-micrometer resolution depending on the master mold quality and process parameters.^65^ This high resolution allows for the precise patterning of microchannels and the integration of complex architectures that closely mimic the *in vivo* microenvironment of human tissues. However, the process involves multiple manual steps, such as PDMS casting, curing, and plasma bonding, which can introduce operator-dependent variability. While soft lithography offers relatively high reproducibility for research-scale production, batch-to-batch consistency may be lower than those with fully automated or industrial-scale techniques.

Bioprinting technology enables the direct deposition of cell-laden bioinks to construct complex tissue architectures within OOC platforms. The achievable resolution in bioprinting is generally lower than that in soft lithography: inkjet and extrusion-based bioprinting typically produce features in the range of tens to hundreds of micrometers, with the finest resolutions possible using advanced light-based (e.g., DLP and two-photon polymerization) strategies. However, when printing with living cells, the resolution is often limited by bioink viscosity, cell size, and viability requirements.^66^ Bioprinting excels at fabricating 3D, multicellular, and tissue-like structures, but reproducibility can be affected by biological variability (e.g., cell sedimentation and bioink stability) and process control. Reproducibility can be improved by automation and standardized protocols, but it remains more variable than that in injection molding due to the biological components involved.

Injection molding technology is commonly used for mass production of microfluidic devices and offers the highest reproducibility among the three methods. Once a hard mold is fabricated (usually from metal or durable plastic), the process can produce thousands of identical chips with minimal batch-to-batch variation. However, the resolution is typically lower than that of soft lithography, with minimum feature sizes generally in the range of 50–100 μm, although precision molding can sometimes achieve finer details.^3^ Injection molding is less suited for rapid prototyping or for the integration of soft, cell-laden materials, but it is ideal for scalable, cost-effective manufacturing of robust device components.

In summary, soft lithography offers the highest resolution with moderate reproducibility (suitable for research and prototyping); bioprinting provides unique advantages for integrating living cells and constructing 3D tissues, but with lower resolution and reproducibility; and injection molding achieves the highest reproducibility and throughput, albeit with lower resolution and limited material flexibility.

## Biomaterials and cells in OOCs

### Biomaterials for OOC application

Biomaterials in OOC systems serve multiple functions: providing structural support, facilitating cell adhesion and proliferation, enabling nutrient and waste transport, and maintaining appropriate mechanical properties that mimic native tissues. Selecting suitable biomaterials requires careful consideration of organ-specific biochemical, mechanical, and structural properties to accurately recapitulate the native microenvironment and promote functional tissue behavior. The selection process should integrate several key parameters. First, mechanical properties should approximate the stiffness of the target tissue, as substrate rigidity strongly influences cell behavior, differentiation, and mechanotransduction signaling—for example, brain tissue exhibits the lowest Young’s modulus, whereas bone requires substantially higher stiffness. Second, the biochemical composition should reflect tissue-specific ECM protein profiles, as the core matrisome—including collagens, proteoglycans, laminins, and fibronectins—varies across organs and directly governs cellular responses. Third, structural features such as porosity and pore size should support cell infiltration, nutrient diffusion, waste removal, and vascularization.^67^^,^^68^^,^^69^

Hydrogels represent the most widely used class of biomaterials in OOC applications due to their high water content and tunable properties. Natural hydrogels such as collagen, fibrin, and hyaluronic acid offer excellent biocompatibility and contain natural cell recognition sites, but may suffer from batch-to-batch variability and limited mechanical tunability.^70^^,^^71^ Synthetic hydrogels including polyethylene glycol (PEG), polyacrylamide, and poly(N-isopropylacrylamide) provide better control over mechanical and chemical properties but may lack inherent bioactivity.^70^^,^^71^

ECM components play crucial roles in providing biochemical cues for cell behavior. Basement membrane extracts like Matrigel and tissue-specific ECM proteins (e.g., laminin for neural applications, and elastin for vascular applications) are commonly incorporated to enhance physiological relevance. However, these materials often present challenges in standardization and reproducibility.^71^

Recently, decellularised-ECM (dECM) hydrogels derived from human or porcine tissues have emerged as organ-specific biomaterial candidates for OOC fabrication. These hydrogels retain native ECM proteins, growth factors, and tissue-specific glycosaminoglycans that regulate cell differentiation and function. Several tissue-derived dECMs have demonstrated improved physiological relevance compared to single-component collagen or Matrigel, enabling tissue-specific phenotypes.^72^^,^^73^^,^^74^ Moreover, dECM hydrogels can be combined with synthetic polymers (e.g., PEG, PVA, or PCL) to enhance mechanical stability and printing fidelity.^75^^,^^76^ Nevertheless, variability in decellularization efficiency and batch composition remains a critical limitation, underscoring the need for standardized proteomic characterization and mechanical calibration for batch release testing.

Synthetic polymers such as PDMS, polystyrene, and COC are frequently used for device fabrication due to their optical transparency, ease of processing, and established manufacturing protocols.^71^ However, PDMS presents significant limitations including absorption of small molecules and proteins, permeability to water vapor, swelling, aging, and chemical sensitivity that compromise its suitability for industrial-scale applications and long-term studies.^77^^,^^78^ COC and cyclic olefin polymer (COP) have emerged as alternatives to PDMS for OOC applications. These medical-grade thermoplastics offer several advantages including very low permeability to oxygen and water vapor, enabling precise control of gas concentrations within microchannels. Unlike PDMS, COC and COP are lipophobic materials that do not exhibit unspecific absorption of small molecules, making them particularly suitable for drug testing and development applications. These materials also demonstrate optical properties with transparency in the visible and near UV range, low birefringence, and high Abbe numbers, making them ideal for microscopy (Table 1).^78^^,^^79^ PMMA represents another candidate for replacing PDMS due to its recyclability, biocompatibility, excellent mechanical properties, and critically, its low permeability to small molecules.^70^ Several elastomeric alternatives have been developed to maintain PDMS-like flexibility while addressing its absorption issues. Styrene-(ethylene/butylene)-styrene (SEBS) copolymer and tetrafluoroethylene-propylene (FEPM) elastomer have been specifically designed to eliminate the absorption of hydrophobic molecules, making them ideal for drug discovery and development applications.^80^^,^^81^

**Table 1:** Absorption/permeability properties corresponding to the representative material for each fabrication method

Fabrication Method	Representative material	Drug absorption (%)	Water absorption/swelling (% mass change)	O_2_ permeability (Barrer)	Notes
Soft lithography	PDMS (polydimethylsiloxane)	40%–90% loss for hydrophobic molecules (e.g., rhodamine B, progesterone, and Nile red); <10% for hydrophilic compounds	∼1%–2% (swelling up to 5%)	600–800	high gas permeability; absorbs small hydrophobic molecules^202^^,^^203^^,^^204^
Bioprinting	PCL (polycaprolactone)	<5%–10% for most hydrophobic compounds; negligible for hydrophilic molecules	<0.5%	10–20	low drug absorption; biocompatible and biodegradable scaffold^35^^,^^205^
Injection molding	COC (cyclic olefin copolymer)	<1%–3% for hydrophobic molecules; negligible for hydrophilic	<0.1%	2–4	low absorption and diffusion; high optical clarity^206^^,^^207^^,^^208^
	COP (cyclic olefin polymer)	<3%	<0.1%	1–3	similar to COC; slightly lower gas permeability; ideal for quantitative assays^207^^,^^208^

## Cell sources and types

Primary human cells provide the highest physiological relevance but are limited by donor availability, variability, and finite lifespan in cultures. Primary hepatocytes, endothelial cells, and epithelial cells are commonly used but require specialized isolation and culture protocols. Patient-derived cells enable personalized medicine applications but introduce additional complexity in standardization.^82^^,^^83^

Human induced pluripotent stem cells (hiPSCs) offer unlimited expansion potential and can be differentiated into various cell types, making them attractive for standardized OOC applications. Recent advances in directed differentiation protocols have enabled the generation of functional cardiomyocytes, hepatocytes, neurons, and other specialized cell types. However, the maturation state of hiPSC-derived cells often remains inferior to their primary counterparts.^84^ HiPSC-derived cardiomyocytes (hiPSC-CMs), for instance, often exhibit fetal-like electrophysiological properties and limited contractile force compared with adult cardiomyocytes; mechanical conditioning, electrical pacing, and perfusion culture systems can partially enhance maturation.^85^^,^^86^^,^^87^^,^^88^^,^^89^

Immortalized cell lines provide reproducibility and ease of handling but may lack physiological relevance due to genetic modifications required for immortalization. Cancer cell lines are frequently used in tumor-on-chip models, while transformed normal cell lines serve as standardized models for basic research.^90^

Co-culture systems in OOC platforms often incorporate multiple cell types to recapitulate tissue complexity. For example, liver-on-chip systems may include hepatocytes, Kupffer cells, stellate cells, and endothelial cells to model the multicellular liver environment.^91^ The optimization of cell ratios, spatial organization, and culture conditions for co-culture systems presents ongoing challenges.^92^

Furthermore, the absence of immune components in many OOC models limits their predictive capability for inflammatory and immuno-toxicological studies. Incorporation of macrophages, dendritic cells, or peripheral blood mononuclear cells (PBMCs) into the liver-, lung-, and vascular-chip models enables the modeling of cytokine signaling, immune cell trafficking, and immunotherapy response. Co-culture with tissue-resident immune cells or the inclusion of patient-derived immune compartments represents a promising direction toward immunocompetent OOC systems, which are increasingly demanded by regulatory agencies for next-generation toxicity and efficacy testing.

## Cultivation methods for OOCs

Living cells and tissues are subjected to a range of mechanical stimuli *in vivo* that play a critical role in defining their specialized functions.^93^ For example, blood vessels surrounding the alveoli of the lung are exposed to shear stress from the flow of blood; moreover, airflow- and stretching-induced shear stresses are also produced on the air-facing side of the alveolar-capillary barrier. This enables the epithelial cells to execute transport functions, including nutrient and oxygen transport. In OOC systems, mechanical stimuli can be reproduced using a pumping or pumpless system and by applying stretch or strain forces.

## Pumping systems

External pumping systems are commonly employed to generate shear flow in microfluidic OOC devices, such as commercial syringe, peristaltic, or pressure-driven pumps,^93^^,^^94^ which include single or multiple inlets and outlets.^95^^,^^96^^,^^97^ These systems support one-pass or recirculatory perfusion flows and enable accurate control of fluid flow rates, gradients, and shear stresses. The selection of the flow type and flow rate (measured as volume per unit time) depends on the requirements of the organ model and the corresponding *in vivo* conditions, including a suitable level of shear stress. Using this system, culture media containing nutrients, growth factors, and signaling molecules are continuously perfused through channels, delivering essential resources to the cells and removing waste products to mimic blood circulation in the human body. Flow rates ranging from 1 to 100 μL are mostly used in OOC systems.^98^

The integration of perfusion sensors into OOC pumping systems enables real-time monitoring and feedback control of fluid flow parameters. Optical fiber-based sensors have proven particularly effective for continuous, non-invasive monitoring of oxygen consumption rates and metabolic activity in liver-on-chip models.^99^ These sensors can be positioned upstream and downstream of tissue chambers to measure differential oxygen consumption, providing direct indicators of tissue viability and metabolic function. The implementation of PID (proportional-integral-derivative) controllers with integrated pressure sensors enables automated maintenance of constant pressure differences and flow rates, significantly improving experimental reproducibility and reducing manual intervention requirements.^100^

Pumping systems in OOC devices are specifically designed to generate and control shear stress levels that mirror physiological conditions. Computational fluid dynamics simulations are commonly employed to predict and optimize shear stress distributions within microfluidic channels, ensuring that the wall shear stress values remain within the physiological range of 0.1–9.5 Pa.^101^^,^^102^^,^^103^ The geometry of microfluidic channels, including the incorporation of micropillars and varying channel widths, can be precisely engineered to create specific shear stress gradients perpendicular to flow direction. Advanced systems can generate both steady-state and pulsatile flow patterns, with pulsatile flows being particularly important for cardiovascular applications where arterial-like flow conditions need to be replicated.^104^ The ability to create controlled shear stress gradients enables researchers to study cellular responses to varying mechanical stimuli within a single chip, providing valuable insights into mechanotransduction processes.

One-pass flows guarantee consistent delivery of fresh nutrients; however, they allow inter-tissue communication only in a single direction.^1^ Recirculatory perfusion flows are not suitable for replacing nutrients and waste products; however, they enable the recirculation of signaling molecules.

## Pumpless systems

Recent advancements of OOCs have led to the removal of external pumps and tubing from their design.^105^^,^^106^^,^^107^ These include systems with the application of hydrostatic pressure and resistance circuit,^108^ gravity-induced bidirectional flow,^39^^,^^109^ thread-driven flow,^110^ and gravity-driven siphon flow.^111^^,^^112^ These passive flow control techniques facilitate the continuous circulation of culture media within the chip and eliminate the requirements for mechanical pumps, thereby reducing the overall complexity of the system. This simplification offers several benefits, including the elimination of intricate pumping mechanisms and related equipment, thereby reducing costs and enhancing user friendliness. Moreover, despite this simplification, these devices effectively replicate the interactions of soluble factors across various organ compartments. This system enables the transfer of original compounds and metabolites across different organs while accommodating a wide range of flow rates, which depend on various factors, including the cross-sectional area of the channel, its overall length, and differences in height.

Despite their overall advantages, pumpless approaches exhibit several common drawbacks when applied to OOC systems. Generally, these systems offer limited adaptability in adjusting flow conditions and do not permit swift changes in the culture medium, such as in the case of drug exposure to cells. Furthermore, the mechanisms for delivering mechanical strain to tissues are often complex, and such systems frequently require custom designs to effectively produce appropriate levels of mechanical stimuli.

Specific pumpless approaches present their own unique challenges. For example, thread-driven flow systems utilize a hydrophilic thread in the outlet of the microfluidic circuit to achieve a continuous medium flow without pumps or external power by leveraging the capillary action of the thread and evaporation in a controlled environment. However, the specific design of these thread-based devices can limit their application in certain OOC systems.^110^

Another widely adopted pumpless solution is the rocking platform, which drives fluid flow in OOC devices by periodically tilting the entire microfluidic device back and forth. This induces gravity-driven bidirectional flow of the culture medium between reservoirs connected by microchannels. While the angle and frequency of rocking can be adjusted to modulate the flow rate and the aspects of physiological recirculation such as blood flow can be effectively mimicked, rocking platforms result in uneven shear stresses across the system and are relatively expensive to implement compared to other passive flow techniques.

### Stretch/strain force-induced system

**Figure 2:**
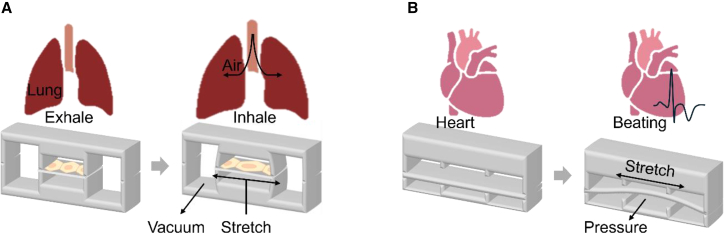
Dynamics of stretch and strain forces in organ-on-a chip models Utilizing either mechanical or pneumatic approaches, these systems replicate the dynamic physical states of (A) lungs and (B) hearts, ensuring precise cellular functionality and behavior.

Mechanical stimulation through stretch and strain forces is essential for replicating the dynamic physical environments experienced by organs *in vivo*. These systems employ either mechanical approaches or pneumatic approaches to simulate physiological movements with precise control over frequency, intensity, and duration to avoid cellular damage while maintaining organ-specific functionality. Lung-on-a-chip models utilize cyclic stretching to emulate breathing motions,^32^ directly influencing alveolar epithelial cell behavior and barrier function. Similarly, heart-on-a-chip platforms incorporate rhythmic strain patterns that are critical for cardiomyocyte contractility and maturation^113^ (Figure 2). The successful implementation of these mechanical cues significantly enhances cell differentiation and provides physiologically relevant organ models, though it increases system complexity due to the need for sophisticated control mechanisms.^94^^,^^114^^,^^115^^,^^116^^,^^117^ Current developments focus on improving system precision and durability while integrating real-time monitoring capabilities. Advanced sensor technologies now enable continuous tracking of applied mechanical forces and the resulting cellular responses, providing valuable insights into mechanotransduction pathways and organ functionality.^18^^,^^118^^,^^119^^,^^120^ These innovations are advancing OOC technology toward more accurate disease modeling and therapeutic screening applications.^114^^,^^121^^,^^122^

## Applications of OOCs

OOC technology represents a paradigm shift in biomedical research and drug development, positioning itself as a critical bridge between traditional *in vitro* methods and animal models. To fully understand the transformative potential of OOCs, it is essential to examine the inherent limitations of conventional approaches and demonstrate how OOCs systematically address these challenges.

Traditional *in vitro* methods, while offering cost effectiveness and high-throughput capabilities, suffer from significant limitations in physiological relevance. Static cell cultures fundamentally fail to replicate the dynamic microenvironments, mechanical forces, and complex cell-cell interactions that are characteristic of living organs. Animal models, despite providing valuable systemic complexity, face substantial translational barriers due to interspecies differences in metabolism, genetic expression, and disease mechanisms. Compelling evidence indicates that up to 90% of drugs showing promise in animal studies ultimately fail in human clinical trials, largely attributed to these fundamental translational discrepancies.^123^^,^^124^

OOC technology systematically addresses these limitations by incorporating human cells into physiologically relevant microenvironments that accurately recapitulate organ-level functions. These advanced models facilitate a comprehensive assessment of drug efficacy, safety, and toxicity within controlled microenvironments that closely mimic human organ physiology, while simultaneously maintaining the experimental control and reproducibility advantages inherent to *in vitro* systems. Through the integration of human cells or tissue constructs into sophisticated microfluidic systems with precise control over fluid flow, mechanical stimulation, and biochemical gradients, OOCs provide substantially more accurate representations of the complex interactions and responses of human organs to various therapeutic compounds.

The strategic positioning of OOCs as potential replacements for traditional methods varies significantly by the application domain. In toxicology screening, OOCs have already achieved notable regulatory recognition, with FDA qualification of liver-on-chip models for DILI assessment representing a significant milestone in regulatory acceptance.^16^ For disease modeling and drug discovery applications, OOCs serve as powerful complementary tools that can substantially reduce reliance on animal testing while providing human-relevant data that traditional cell cultures simply cannot achieve. However, complete replacement of animal models remains a long-term objective due to current technological limitations in fully replicating systemic complexity, comprehensive organ-organ interactions, and complete immune system integration.^11^^,^^12^

The most comprehensive validation study demonstrated that the OOC technology could achieve 87% sensitivity and 100% specificity for DILI prediction,^125^ compared to animal models that correctly predict human outcomes only about 50% of the time-essentially random chance.^126^ This landmark Emulate study analyzed 870 liver-on-chip models across 27 blinded hepatotoxic and non-toxic drugs, establishing a Spearman’s correlation of 0.78 with clinical DILI severity scales.^125^ Traditional hepatocyte cultures and 3D spheroids performed significantly worse, achieving only 42%–65% sensitivity and 67% specificity.^127^ High-throughput screening platforms like OrganoPlate LiverTox demonstrated better predictive sensitivity than all previously reported *in vitro* models when testing 159 known hepatotoxic compounds.^128^

Kidney-on-chip models have demonstrated capability for elucidating mechanisms of drug-induced nephrotoxicity and identifying therapeutic interventions. Cohen et al. (2021) developed sensor-integrated vascularized kidney spheroid-on-chip platforms that successfully identified glucose accumulation as the mechanistic driver of cyclosporine- and cisplatin-induced nephrotoxicity. Their platform demonstrated that impeding glucose reabsorption using sodium-glucose cotransporter 2 (SGLT2) inhibitors blocked cyclosporine and cisplatin toxicity by 1000-fold and 3-fold, respectively. These findings were validated through a retrospective clinical study of 247 patients (113 men and 134 women, with average age of 48 years) who were diagnosed with kidney damage while receiving cyclosporine or cisplatin. Patients taking cyclosporine or cisplatin alone showed elevated serum creatinine, uric acid, and lactate dehydrogenase (LDH) levels, with decreased estimated glomerular filtration rates (eGFR) indicating kidney damage. However, patients receiving combination therapy with the SGLT2 inhibitor empagliflozin showed significant (*p* < 0.001) improvement in kidney function, with the eGFR rising above 90 mL/min per 1.73 m^2^ and substantial reduction in serum creatinine, uric acid, and LDH levels. These clinical results directly validated the kidney-on-chip mechanistic predictions, demonstrating the translational value of the platform.^129^ Lin et al. (2019) reported 3D vascularized proximal tubule models that quantitatively reproduced renal reabsorption functions. These models demonstrated nearly 5- to 10-fold enhancement in glucose reabsorption compared with 2D transwell controls, with the 3D vascularized proximal tubule (VasPT) tissue achieving steady-state reabsorption of approximately 11 μg glucose/mm^2^ per day. The platform successfully recapitulated selective albumin uptake via receptor-mediated endocytosis while maintaining barrier integrity against inulin transport, validating tubular-vascular exchange mechanisms characteristic of native kidney tissue architecture. Importantly, the engineered tissues demonstrated approximately 20% of native kidney glucose reabsorption efficiency (∼11 μg glucose/mm^2^ in the chip model versus ∼60 μg glucose/mm^2^ in healthy human kidneys *in vivo*), representing a substantial advancement for *in vitro* nephrotoxicity screening and mechanistic studies.^42^

Blood-brain barrier (BBB)-on-chip platforms have demonstrated quantifiable barrier function and drug permeability assessment capabilities essential for CNS drug development. Wevers et al. (2018) developed a perfused human BBB-on-chip in high-throughput format within a 384-well plate housing 96 or 40 chips. The platform integrated human brain endothelial cells, astrocytes, and pericytes in a membrane-free microfluidic system under perfusion conditions. The BBB-on-chip demonstrated functional barrier integrity by severely limiting the passage of a 20 kDa FITC-dextran dye, showing presence of adherens and tight junctions including claudin-5, VE-cadherin, and PECAM-1 at cell-cell contacts. The platform successfully tested permeability profiles of more than 20 therapeutic antibodies and small molecule drugs, demonstrating sufficient sensitivity to distinguish between receptor-mediated transcytosis pathways. When perfused with an anti-transferrin receptor antibody (MEM-189), the platform showed apparent permeability (P app) of 2.9 × 10^−5^ cm/min, markedly higher than that for the control antibody (1.6 × 10^−5^ cm/min). This differential permeability demonstrates the platform’s capability to identify and quantify receptor-mediated transport mechanisms critical for predicting CNS drug penetration.^130^

Herland et al. (2020) demonstrated quantitative human pharmacokinetic predictions by using fluidically coupled multi-organ platforms integrating gut, liver, and kidney chips. Their body-on-chip system achieved a high pharmacokinetic correlation with published human clinical data for both orally administered nicotine and intravenously administered cisplatin, with Lin’s concordance coefficients rated as “good to very good” and Pearson’s correlation coefficients reaching 0.99 for cisplatin in the arteriovenous reservoir. Critically, the platform accurately predicted not only drug absorption and clearance kinetics but also organ-specific toxicity patterns and metabolite formation profiles, demonstrating the translational potential of interconnected organ systems.^131^

Despite these advances, current OOC technology faces significant limitations that must be acknowledged. The liver-chip systems maintained a coefficient of variation below 5% for urea production on day 1, increasing to 20% by day 7 across different hepatocyte donors,^125^ highlighting ongoing challenges with donor-to-donor variability in primary human cells. Intra-laboratory reproducibility studies showed that most OOC assays demonstrated “acceptable” to “excellent” correlation within studies,^132^ but inter-laboratory standardization remains a challenge due to variations in cell sources, culture protocols, and analytical endpoints. Patient-derived cells, while offering advantages for personalized medicine applications, introduce additional complexity in standardization and require careful validation against clinical outcomes.^82^

Endpoint harmonization represents another critical limitation. The lack of standardized readouts, acceptance criteria, and validation protocols across different OOC platforms complicates cross-study comparisons and regulatory acceptance. While the FDA’s qualification of liver-chip models for DILI assessment through the ISTAND program represents a significant milestone,^16^ similar regulatory frameworks for other organ systems remain under development. Ongoing efforts by the international standard organizations and industry consortia aim to establish common metrics, quality control procedures, and reporting guidelines to facilitate broader adoption and regulatory acceptance.

Furthermore, current OOC platforms cannot yet fully recapitulate the systemic complexity present in living organisms. Key limitations include incomplete immune system integration, absence of neuroendocrine feedback loops, and challenges in maintaining long-term organ-organ interactions at physiologically relevant scales.^11^^,^^12^ Most current systems lack functional adaptive immune components, limiting their ability to model immune-mediated toxicity, inflammatory responses, and immunotherapeutic mechanisms. The integration of comprehensive immune cell populations, including circulating lymphocytes, tissue-resident macrophages, and adaptive immune responses, remains an active area of development.^12^

Consequently, OOC technology has emerged as a truly transformative tool for commercial testing, revolutionizing the preclinical evaluation of drugs, therapeutics, and other products by offering significant advantages over traditional *in vitro* and animal testing methods through substantially more physiologically relevant platforms.^19^^,^^125^^,^^133^ However, these systems should currently be viewed as powerful complementary tools that can substantially reduce reliance on animal testing while providing human-relevant data, rather than complete replacements for all preclinical testing paradigms. Complete replacement of animal models remains a long-term objective that will require continued technological innovation, extensive validation across therapeutic areas, and establishment of comprehensive regulatory frameworks.

## Commercial testing

A primary application in commercial testing is in the field of drug development. OOC models evaluate drug efficacy by replicating physiological conditions and disease mechanisms relevant to specific organs. These models enable the assessment of drug absorption, distribution, metabolism, and excretion in a more realistic context, providing valuable insights into drug behavior and therapeutic potential. Moreover, by simulating the physiological environment of organs, these chips can replicate disease states at the cellular level, thereby providing invaluable insights into disease mechanisms and potential therapeutic targets. This allows OOCs to play a crucial role in the burgeoning field of personalized medicine, paving the way for more personalized treatment plans and reducing the risk of adverse drug reactions.

The US FDA signed a collaborative agreement with Emulate, Inc. to use their “human emulation system,” a commercial OOC system, for advanced toxicity testing.^134^ This system aims to enhance the understanding of the impact of various products on human health and safety. Designed to mimic the physiological conditions of specific human tissues and organs, this system offers a more accurate and detailed predictive model of human responses to diseases, medications, chemicals, and food than traditional preclinical testing methods, such as cell cultures or animal experiments. The proprietary Organ-Chips developed by Emulate, Inc., which include models for the lungs, liver, brain, and kidneys, are integral to this system. Each chip, which is approximately the size of an AA battery, features channels lined with living human cells and tissues, providing a dynamic micro-engineered environment that replicates the natural physiological and mechanical forces experienced by cells in the human body. This innovation offers researchers real-time insights into human biology and disease, predicting human responses with greater precision than current cell culture or animal-based testing methods. The Liver-Chip developed by Emulate represents the first OOC technology accepted into the FDA’s ISTAND Pilot Program in September 2024. This acceptance marks a critical milestone in regulatory recognition of microphysiological systems (MPSs) as legitimate drug development tools. The liver-chip was accepted for a specific context of use: assessing the relative risk of DILI in adult patients by comparing candidate drugs to structural analogs from the same therapeutic class.^16^ Emulate formed a partnership with Roche to use Organ-Chips for testing the efficacy and safety of new antibody therapeutics and combination therapies. This collaboration specifically focuses on lung-chips and brain-chips to gain insights into disease mechanisms and increase the predictability of biomarkers. Similarly, the company partnered with Takeda Pharmaceutical to use their Intestine-Chip for gastrointestinal (GI) disease drug discovery, representing the first application of this technology to GI disease research.^135^^,^^136^ Obatala Sciences has launched a service utilizing their ObaCell Obesity-on-a-Chip, which uniquely replicates the physiology of obese patients in a laboratory setting. This innovation overcomes previous limitations in culturing mature adipocytes in a dish and enables the simulation of fasting and feeding conditions in a human model system.^137^ MIMETAS has achieved the most widespread pharmaceutical industry adoption of any OOC platform according to publication analysis. The company’s OrganoPlate technology features standard microplate formats (40-well, 64-well, and 96-well configurations) that integrate seamlessly with existing pharmaceutical laboratory infrastructure, including automated liquid handlers, robotic systems, and plate readers. This compatibility addresses a critical adoption barrier: pharmaceutical R&D facilities have invested heavily in automation equipment designed for standard microplate formats, and technologies that deviate from this format face significant implementation friction. The OrganoPlate’s three-lane configurations enable direct access to both apical and basolateral sides of cultured tissues, facilitating perfusion studies and barrier integrity assays that are essential for ADME-Tox (absorption, distribution, metabolism, excretion, and toxicity) profiling. This design allows pharmaceutical researchers to assess bidirectional transport, evaluate efflux transporter activity, and measure tight junction integrity—parameters that are critical for predicting human oral bioavailability and tissue distribution. Major partnerships include collaborations with Roche to develop intestinal barrier models for evaluating drug permeability and absorption, and with multiple pharmaceutical companies for inflammatory bowel disease (IBD) and hepatitis B virus (HBV) modeling.^138^^,^^139^ In addition to this, numerous OOCs have been released till now (Table 2). These systems enable researchers to generate better data during the preclinical research phase of drug development, resulting in shorter timelines and better treatments.

**Table 2:** Overview of marketed organ-on-a-chip products and their manufacturers for tissue-based drug evaluation^209^^,^^210^

Company	Main/Key material	Features	Products	Reference
Emulate	PDMS	• replication of human biology (e.g., air-liquid interface) • automating the precise condition by using its own module • simplification of data analysis	Brain-Chip Colon Intestine-Chip Duodenum Intestine-Chip Kidney-Chip Liver-Chip Lung-Chip	Villenave et al.^211^ Workman et al.^212^ Sances et al.^213^ Peel et al.^214^ Nawroth et al.^215^
Alveolix	collagen-elastin, PDMS	• ultrathin membrane • breathing motion	^AX^Barrier-on-Chip System ^AX^Lung-On-Chip System	Stucki et al.^216^^,^^217^
Mimetas	polystyrene	• membrane-free tissue culture • perfusion without pumps and tubing	OrganoReady® Colon Caco-2^a^ OrganoReady® BBB HBMEC^a^ OrganoReady® Blood Vessel HUVEC^a^ OrganoReady® Angiogenesis HUVEC^a^	Morelli et al.^218^ Wevers et al.^130^ Ehlers et al.^219^ Camilla Soragni et al.^220^
Aim biotech	cyclic olefin polymer	• various design option (2D to large 3D tissue)	organiX idenTx	Hajal et al.^221^ Pars et al.^222^
Obatala Sciences	well plate format with human-derived hydrogels, plastic (unkwon)	• including human-derived hydrogels, human stem cells, and supporting cell culture mediums	ObaCell® Adipose-On-A-Chip	McCarthy et al.^223^
Curi bio	magnet, plastic (unkwon)	• structurally mature and predictive human engineered cardiac, skeletal muscle, and disease models	Mantarray™	Smith et al.^154^
Organovo	various plastics and hydrogel (e.g., NovoGel)	• enable fabricating scaffold-free tissue • not restricted by shape	ExVive™	Irelan et al.^224^
CN-BIO	cyclic olefin copolymer	• PDMS-free plates reduce non-specific binding • large sampling volumes facilitate highly multiplexed endpoint analysis	PhysioMimix®	Novac et al.^225^ Milani et al.^226^

a Caco-2, human Caucasian colon adenocarcinoma cell line; HBMEC, human brain microvasculature endothelial cell; HUVEC, human umbilical vein endothelial cell.

One of the most significant barriers to pharmaceutical industry adoption is the lack of standardized quality control (QC) protocols and batch release criteria for the OOC systems. Unlike traditional cell-based assays, which rely on decades of well-defined QC metrics, organ-chips incorporate multiple primary or stem cell-derived cell types, ECM components, and microfluidic hardware, creating multi-parameter systems in which quality can vary at several levels. Current QC challenges include: (1) cell-source variability, as primary human cells exhibit substantial donor-to-donor heterogeneity in phenotype, metabolic function, and stress responses; (2) batch-to-batch variation in ECM materials—particularly Matrigel—which displays an inconsistent protein composition and growth factor content across manufacturing lots; (3) fabrication variability in microfluidic devices that alters channel geometry, surface chemistry, flow resistance, and mechanical properties; and (4) the absence of harmonized functional qualification assays capable of verifying barrier integrity, metabolic competence, or immune responsiveness for each chip prior to experimental use. To meet industrial expectations, OOC platforms require clearly defined batch release criteria—quantitative acceptance thresholds for structural, biological, and functional performance metrics that determine whether a manufactured batch is suitable for toxicology or efficacy testing. Establishing such QC frameworks is now considered a critical unmet need for scalable pharmaceutical adoption of OOC technologies.^140^^,^^141^^,^^142^^,^^143^

### Biological research

#### Disease modeling

OOCs effectively replicate tissue-level pathological features, aiding in accurate drug identification. For instance, a pulmonary edema-on-a-chip model was developed by introducing interleukin-2 into the microvascular channel of a lung-on-a-chip, which comprises alveolar and microvascular channels.^144^ Interleukin-2 induction led to pulmonary leakage and simulated edema. In response, the drug GSK2193874 was applied, which successfully inhibited this leakage, demonstrating the potential of OOCs in drug testing and disease modeling. Conversely, OOCs demonstrate significant promise in accurately recreating the complex environment of tumors.^41^^,^^145^^,^^146^^,^^147^^,^^148^ Studies have shown a correlation between patients and OOCs of blood vessels and various cancers such as glioblastoma,^41^ breast cancer,^148^ lung cancer,^145^^,^^147^ and gastric cancer,^146^ enabling more precise drug testing. Moreover, the gastric cancer OOC was used to recapitulate the pathological features of patients, which were evaluated through transcriptomic analysis and histological examination.^146^ This approach has successfully reproduced clinical drug response reproducibility, showcasing the potential of OOCs in personalized medicine and cancer research.

Moreover, multi-OOCs integrate various types of tissues/organs for investigating the communications between tissues/organs and metabolic pathways of drugs through multiple tissues/organs.^13^^,^^131^^,^^149^ Trapecar et al. interconnected the MPSs of the human gut, liver, and brain to study neurodegenerative diseases.^150^ The interactions within the system enhanced the *in vivo*-like behavior of cerebral MPSs. Moreover, short-chain fatty acids associated with the microbiome increased the expression of pathways linked to Parkinson disease. Kim et al. developed an integrated OOC model comprising pancreatic, liver, and visceral adipose tissues to simulate the pathology of type 2 diabetes. This model revealed that organ interactions significantly elevated metabolite levels, with proteins such as insulin, tumor necrosis factor alpha, albumin, and interleukin-6 showing higher concentrations in the integrated chip relative to those in single- or dual-organ setups. The model effectively demonstrated the breakdown of glucose metabolism owing to the combined effect of hyperglycemia and inflammation, which are characteristic of type 2 diabetes.^151^

These models enable the study of disease initiation, progression, and treatment response in controlled and dynamic microenvironments. By integrating disease-specific cells or tissues, OOC devices contribute to a better understanding of disease mechanisms and the development of targeted therapies.

### Biosensor-integrated OOCs

The integration of biosensors into OOC technology has led to significant advancements in biological research. These biosensor-integrated OOCs enhance the real-time monitoring and analysis of physiological responses at the cellular level.^152^ They facilitate the continuous measurement of various biomarkers, cellular activities, and drug responses, providing a more dynamic and comprehensive understanding of biological processes. Yong et al. created a tissue-sensor platform, using biohybrid 3D printing methods,^153^ enabling wireless real-time monitoring of drug-induced cardiotoxicity. This innovative platform incorporates a strain-gauge sensor and engineered heart tissue (EHT), using a variety of inks, including conductive and biocompatible materials. It enabled the continuous monitoring of the contractile force of the EHT, providing insights into the acute and chronic effects of cardiotoxic substances. This advancement is a significant contribution to biological research, particularly in drug testing and cardiotoxicity studies, and offers a more dynamic and precise approach than that of traditional methods. The real-time data collection offered by these biosensors within OOCs enables more precise and accurate studies, thereby significantly contributing to the advancement of biomedical research and therapeutic development. Smith et al. developed a high-throughput, real-time monitoring system for engineered skeletal muscles that utilizes magnetic sensing.^154^ This technology facilitates the non-invasive and longitudinal analysis of muscle function, providing insights into contractility and response to pharmacological agents. Son et al. engineered a cerebrospinal assembly system integrating brain organoids and motor neuron spheres to investigate cortical output signals and spinal cord transmissions. In this platform, cortical signals and spinal transmission were monitored using a multi-electrode array.^155^ Lee et al. fabricated an integrated chip platform combining heart and breast cancer components and detected biomarkers related to chemotherapy-induced cardiotoxicity by using immuno-aptasensors.^156^ Such advancements of OOC technology that integrate sophisticated biosensors offer significant improvements in drug screening, disease modeling, and the understanding of physiology, paving the way for more accurate and efficient biomedical research.

Beyond the examples mentioned above, several specific biosensing modalities have demonstrated particular utility in OOC systems. Electrochemical *trans*-endothelial electrical resistance (TEER) measurement has become a gold standard for real-time assessment of barrier integrity in organ models. Integrated TEER electrodes enable continuous, non-invasive monitoring of tight junction formation and barrier function in endothelial-epithelial barriers.^157^^,^^158^ These systems typically employ microelectrode arrays positioned on either side of a porous membrane, measuring impedance across the cellular barrier at physiologically relevant frequencies. Modern TEER-integrated chips can detect barrier disruption within minutes of toxin exposure, providing early warning signals that are likely to be missed by endpoint assays.

Fluorescent metabolic sensors represent another powerful class of integrated biosensors. Oxygen-sensitive fluorophores enable real-time measurement of oxygen consumption rates, a critical indicator of cellular metabolic activity and mitochondrial function.^99^^,^^159^^,^^160^ Similarly, pH-sensitive probes allow continuous monitoring of acidification and lactate production, while genetically encoded reporters including GCaMP and ATP sensors visualize intracellular signaling with subcellular resolution.^18^^,^^159^^,^^161^^,^^162^^,^^163^ These fluorescent readouts can be multiplexed with brightfield imaging to correlate metabolic state with morphological changes.

However, the integration of biosensors into OOC systems presents several significant challenges that must be addressed to ensure reliable long-term monitoring. Signal drift represents a persistent problem, particularly for electrochemical sensors, where electrode fouling, protein adsorption, and changes in reference electrode potential can cause baseline shifts over multi-day culture periods.^118^^,^^164^ Strategies to mitigate drift include the use of differential measurements with on-chip reference chambers and the implementation of self-cleaning electrode surfaces.^165^^,^^166^^,^^167^

Data standardization and harmonization remain underappreciated obstacles to the widespread adoption of biosensor-integrated OOCs. Different sensor platforms often produce outputs in arbitrary units (e.g., fluorescence intensity and impedance magnitude) that are difficult to be compared across laboratories or converted to physiologically meaningful concentrations. The lack of consensus on calibration protocols, quality control metrics, and data reporting formats hinders reproducibility and meta-analysis. The community must establish agreed-upon benchmarks, reference materials, and inter-laboratory proficiency testing to ensure that biosensor data from OOC systems can be reliably interpreted and compared across studies.

## OOCs in space

**Figure 3:**
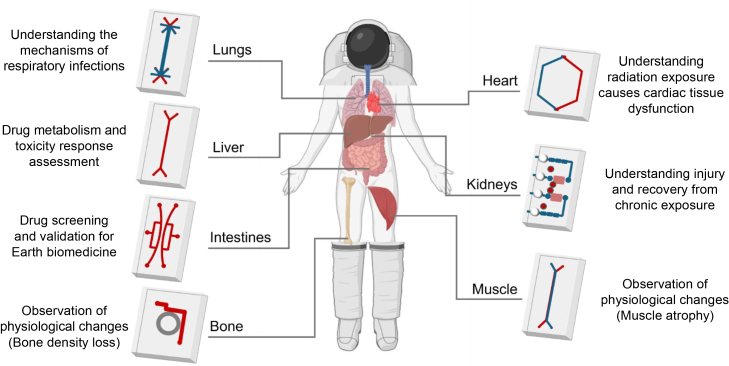
Tissue chips in space Overview of tissue chip designs aimed at emulating a variety of tissue-level physiological activities, incorporating microchannels and built-in microstructures to study the biological impacts of spaceflight conditions such as microgravity and cosmic radiation.

Furthermore, OOCs are currently being tested in space stations to study the biological behavior of human tissues that are impacted by various factors occurring in space. Recently, OOC systems were sent to the International Space Station (ISS) by the National Aeronautics and Space Administration and other agencies to study the effects of microgravity^168^^,^^169^ and space radiation.^170^ Microgravity causes changes in various organs and tissues, including the immune, musculoskeletal, renal, and cardiac systems.^168^ Moreover, space radiation poses risks, such as DNA damage,^171^ cancer,^172^ and other health issues.^173^^,^^174^ OOC studies in space are crucial for understanding and addressing these unique challenges (Figure 3).

In 2006, the growth and metabolic activity of microorganisms were monitored under microgravity in low Earth orbit, using a “nanosatellite.”^175^ In 2019, OOC models imitating the lungs, bone marrow, bones, cartilages, kidneys, and BBB were sent to the ISS.^176^ This mission aimed to enhance the understanding of the effects of microgravity on human health and diseases. Subsequently, in 2020, intestinal and heart chips were sent to the ISS. Furthermore, ongoing tests on the ISS using OOCs are studying the causes of muscle wastage and investigating immune aging and healing outcomes. These efforts are vital for comprehending the physiological changes in astronauts and have wider implications for medical research on Earth, particularly in understanding disease mechanisms and treatments under conditions such as microgravity and space radiation (Table 3).

**Table 3:** NASA-funded multi-organ tissue chip projects for deep space exploration and aging research^227^

Targeted Work	Objective	Approach	Application	Team (institution)
Brain-liver-gut axis	model multicellular tissue responses to spaceflight stress and aging	enhancing the durability of a multi-organ system involving the brain, liver, gut, and immune components	enable TIDES; understand degenerative diseases and aging	Elizabeth Blaber, Rensselaer Polytechnic
Brain	analyze genetically diverse brain responses to neurotoxic stress	extending the longevity of miBrain for studying response of human brain tissue to neurotoxic stress	identify biomarkers and mechanisms of spaceflight-induced brain disorders	Joel Blanchard, Icahn School of Medicine at Mount Sinai
Neurovasculature	create a long-lasting 3D neurovascular	long-term (1–6 months) 3D human brain vasculature model with ECs, pericytes, and astrocytes under chronic stress	evaluate chronic inflammation and neurodegeneration	Guohao Dai, Northeastern
Anthracycline vascular	investigate doxorubicin-induced vascular dysfunction	flow-conditioned vascular model for long-term (at least 6 months) doxorubicin response analysis	develop therapies for off-target drug effects	Guillermo García-Cardeña, Brigham and Women’s Hospital
Atherosclerosis	use vessel chips from patient iPSCs to study vascular disease	physiologically relevant microfluidic co-culture for studying radiation- and age-related vascular diseases	identify radiation and aging-related vascular therapies	Abhishek Jain, Texas A&M
OTE: Organ tissue equivalents	model responses of single and multi-organ tissue to stressors	microengineered 3D tissue equivalents of 12 human organs (cardiac, brain, bone marrow, lung, liver, testis/ovary, intestine, skin, and vasculature) using primary cells	understand tissue-specific reactions to spaceflight	Christopher Porada, Wake Forest University
MORPH: multi-organ repair post hypoxia	study organ recovery from acute hypoxia	multi-organ platform with vascular linkage and brain integration for extended viability testing	protect critical systems like brain, heart, bone marrow	Gordana Vunjak-Novakovic, Columbia University
Engineered heart and vasculature	use engineered heart/vascular tissues to study radiation effects	iPSC-derived cardiac and vascular-like engineered tissues	clarify disease pathways from radiation exposure	Joseph Wu, Stanford University
Kidney	extend kidney MPS/organoid culture for chronic toxicity models	prolonged modeling (up to 6 months) of stress exposure and recovery, using kidney MPS and organoids	model long-term drug/toxin injury and recovery	Catherine Yeung, University of Washington

The deployment of OOC systems in space presents significant engineering challenges that require complete re-engineering of ground-based platforms.^177^ The extreme physical space limitations on the ISS necessitate dramatic system miniaturization, with equipment volumes reduced from approximately 1,350 L on Earth to just 45 L for installation on the space station. This compression requires the elimination of flexible tubing, complete redesign of perfusion and environmental control systems, and development of self-contained modules that house motors, temperature/CO_2_/humidity control, and media cassettes.^177^ The engineering teams must create entirely novel pumping and incubation systems while maintaining proper environmental conditions for cell culture.^178^

The absence of gravity fundamentally alters fluid dynamics within microfluidic channels, creating unique engineering obstacles. Surface tension becomes the dominant force governing fluid behavior, while buoyancy-driven convection is eliminated.^179^^,^^180^ This leads to altered flow patterns and bubble formation challenges that can significantly disrupt OOC functionality.^181^^,^^182^ Bubble formation represents a critical technical challenge, as even microscopic bubbles can accumulate and grow, causing highly disruptive fluid dynamics that completely interrupt the low-shear environment essential for proper cell culture.^182^ The absence of gravitational forces also affects the transport of fluids through porous structures, with gravity exerting a significant effect on flow patterns through microfluidic features.^179^

Microgravity profoundly affects cellular behavior, creating challenges for maintaining physiologically relevant OOC models.^183^ Cells exposed to microgravity exhibit altered proliferation patterns, with embryonic stem cells showing stronger proliferative characteristics and longer survival probability than ground controls.^183^ However, microgravity inhibits effective terminal differentiation, with cells maintaining a high expression of early differentiation markers, even after extended culture periods.^183^ This altered differentiation capacity poses significant challenges for creating mature, functional tissue models in space-based OOC systems. Microgravity also affects the integrity of biological barriers critical for organ function, including the BBB and epithelial barriers. Changes in tight junction proteins and barrier integrity have been observed in response to microgravity conditions, potentially compromising the physiological relevance of barrier-dependent OOC models.^178^ This dysfunction can lead to altered permeability, modified immune responses, and disrupted cellular communication pathways that are essential for proper organ function.

The microgravity environment promotes the formation of 3D cell aggregates, rather than the monolayer growth patterns typical of Earth-based culture.^183^ While this 3D growth can be advantageous for certain applications, it creates challenges for maintaining consistent cell distribution and proper tissue architecture within OOC devices.^183^ Cells tend to self-assemble slowly based on cell-to-cell contact and physiologic affinity, rather than being distributed in anatomically correct patterns.^177^ This altered spatial organization can significantly impact the physiological relevance of OOC models and requires careful consideration in experimental design.

## Conclusions and future perspectives

OOC technology has emerged as a transformative approach in biomedical research, offering a powerful platform for replicating the complexity and functionality of human organs in a controlled microenvironment.^2^ Through the integration of microfluidics, 3D printing, and other advanced fabrication methods, OOC devices enable the cultivation of cells and tissues that closely mimic native organs, thereby providing a more physiologically relevant model for studying human biology, disease mechanisms, and therapeutic interventions. OOC technology exhibits significant advantages over conventional models. These innovative devices offer improved accuracy, reproducibility, and scalability compared to traditional *in vitro* systems and animal models. The ability to replicate the dynamic microenvironments, cellular interactions, and physiological processes of human organs provides researchers with powerful tools to gain deeper insights into organ function, disease progression, and drug responses. Applications of OOC technology span a wide range of fields. In commercial testing, OOC models enable more accurate and predictive assessments of drug efficacy, safety, and toxicity, leading to improved decision-making during the drug development process. In biological research, these devices contribute to the study of disease mechanisms, personalized medicine, stem cell research, and advancing our understanding of human physiology. Furthermore, the versatility of OOC technology enables the integration of multiple organs within a single device, facilitating the investigation of organ-organ interactions and systemic responses. This capability holds promise for unraveling complex disease processes, studying organ-specific phenomena, and advancing the fields of pharmacology, toxicology, and regenerative medicine.

As OOC technology continues to evolve, challenges such as scalability, accurate vascularization (e.g., flow, tissue-specificity, co-culture, and maintenance), long-term stability, and standardization of protocols must be overcome.^1^^,^^140^^,^^184^^,^^185^^,^^186^^,^^187^^,^^188^^,^^189^ Ongoing research and technological advancements are rapidly addressing these limitations, paving way for the broader adoption and commercialization of OOC devices.

International standardization efforts for OOC technologies have been advancing through coordinated initiatives that provide actionable guidance for researchers, industry, and regulators. The CEN/CENELEC Focus Group on Organ-on-Chip has developed a comprehensive roadmap covering five key domains: terminology and ecosystem interdependencies; biosciences; engineering aspects; experimental design and data management; and user perspectives with regulatory, legal, and ethical considerations. These activities are aligned with ISO/TC 276/SC 2, which is developing harmonized international standards for OOC systems, and with ASTM International, which has established foundational terminology through ASTM F3570-22. Complementing these efforts, the IQ Consortium’s MPS Affiliate has issued guidelines for developing, qualifying, and implementing complex *in vitro* models during drug discovery, emphasizing context-of-use definition, model characterization, and fit-for-purpose validation criteria. The roadmap also outlines the minimum reporting requirements for biological components (cell-source documentation, QC data, biomaterial characterization, and culture conditions) and experimental design elements (control strategies, sample-size justification, and FAIR-aligned data management). Regulatory qualification pathways include the US FDA’s ISTAND program for New Alternative Methods and the EMA’s scientific advice mechanism for voluntary data submission. Together, these standardization frameworks and reporting checklists aim to ensure reproducibility and comparability across platforms, thereby accelerating regulatory confidence and adoption of OOC technologies in pharmaceutical development, toxicology testing, and personalized medicine.^11^^,^^125^^,^^141^^,^^143^^,^^190^^,^^191^^,^^192^^,^^193^^,^^194^ In parallel, regulatory agencies—most notably the FDA—are establishing broader evaluation frameworks for OOC-based methods. The Predictive Toxicology Roadmap specifically highlights OOC systems as key tools for improving human-relevant safety assessment.^195^ Continued collaboration among researchers, regulators, and industry stakeholders will be essential to establish clear guidelines and acceptance criteria for future OOC-based studies.

The convergence of artificial intelligence (AI) with OOC technology represents one of the most promising frontiers in biomedical research. AI integration offers transformative capabilities across multiple dimensions of OOC development and operation. Machine learning algorithms can optimize experimental design parameters in real time, automatically adjusting the microfluidic flow rates, chemical gradients, and environmental conditions to achieve optimal cellular responses.^196^ Deep learning models have demonstrated success in analyzing the vast datasets generated by OOC systems, identifying subtle patterns in cellular behavior and drug responses that would be impossible for human researchers to discern.^197^^,^^198^ Predictive modeling represents another application of AI in OOC systems. Advanced algorithms can forecast drug toxicity, efficacy, and metabolic pathways before physical experiments are conducted, dramatically reducing the time and cost associated with drug discovery.^196^

Modern high-throughput screening systems integrate robotic liquid handling, advanced analytical techniques, and sophisticated computing to enable simultaneous testing of thousands of compounds across multiple organ models.^199^^,^^200^ These platforms combine the physiological relevance of OOC technology with the scalability required for industrial drug development applications. Integration of robotic systems enables continuous, unattended operation for extended periods, facilitating long-term studies of chronic drug responses and disease progression. The incorporation of automated imaging systems and real-time data collection capabilities allows for comprehensive monitoring of experimental parameters without manual intervention.^18^

The integration of sophisticated biosensing capabilities into OOC platforms has revolutionized real-time monitoring and analysis of physiological responses. Next-generation biosensor technologies should enable continuous, non-invasive measurement of multiple parameters, including oxygen consumption, pH levels, metabolite concentrations, tissue behavior and biomarker expression, simultaneously.^201^ These integrated sensing systems provide dynamic, time-series data that captures the temporal evolution of cellular responses to therapeutic interventions.

Overall, the OOC technology is redefining biomedical research, offering a more physiologically relevant and versatile platform for studying human organs. These innovative devices have the potential to accelerate drug discovery, improve safety testing, enhance our understanding of disease mechanisms, ultimately contributing to the development of personalized medicine. The continuous advancement of the OOC technology holds immense promise for transforming the future of biomedical research and revolutionizing healthcare practices. Furthermore, the microfluidic channels of different organs are interconnected to replicate the transport of nutrients, metabolites, and signaling molecules between organs, simulating the physiological crosstalk observed in the human body.

## Acknowledgments

This work was supported by the 10.13039/501100001321National Research Foundation of Korea grant funded by the Korea government (MSIP) (NRF-2022R1A2C3004300). This research was also supported by Korean Fund for Regenerative Medicine by the Ministry of Science, ICT, and Ministry of Health and Welfare (22A0106L1, Republic of Korea).

## Author contributions

Investigation, J.S.K. and J.K.; visualization, J.K.; writing – original draft, J.S.K. and J.K.; writing – review & editing, J.S.K., J.K., J.J., and D.-W.C.; funding acquisition, J.J. and D.-W.C.

## Declaration of interests

The authors declare no competing interests.

## Declaration of generative AI and AI-assisted technologies in the writing process

During the preparation of this work, the authors used ChatGPT to improve the readability and language of the manuscript. After using this tool, the authors reviewed and edited the content as needed and take full responsibility for the content of the published article.
